# Structural Variability of Lipoarabinomannan Modulates Innate Immune Responses within Infected Alveolar Epithelial Cells

**DOI:** 10.3390/cells11030361

**Published:** 2022-01-21

**Authors:** Hanrui Liu, Xuwen Gui, Shixing Chen, Weizhe Fu, Xiang Li, Tingyuan Xiao, Jie Hou, Tao Jiang

**Affiliations:** 1 Department of Biotechnology, The College of Basic Medical Science, Dalian Medical University, Dalian 116044, China; llliuhanrui@163.com (H.L.); guixvwenn@163.com (X.G.); fuwz123f@163.com (W.F.); lixiangdoc18@163.com (X.L.); txiao@g.emporia.edu (T.X.); houjie@dmu.edu.cn (J.H.); 2 Key Laboratory of Science and Technology on Microsystem, Shanghai Institute of Microsystem and Information Technology, Chinese Academy of Sciences, Shanghai 200050, China; sxchen@mail.sim.ac.cn

**Keywords:** *Mycobacterium smegmatis*, EmbC, lipoarabinomannan, LC3-associated phagocytosis, alveolar epithelial cells

## Abstract

*Mycobacterium tuberculosis* (*M. tb*) is an intracellular pathogen persisting in phagosomes that has the ability to escape host immune surveillance causing tuberculosis (TB). Lipoarabinomannan (LAM), as a glycolipid, is one of the complex outermost components of the mycobacterial cell envelope and plays a critical role in modulating host responses during *M. tb* infection. Different species within the *Mycobacterium* genus exhibit distinct LAM structures and elicit diverse innate immune responses. However, little is known about the mechanisms. In this study, we first constructed a LAM-truncated mutant with fewer arabinofuranose (Ara*f*) residues named *M. sm*-ΔM_6387 (*Mycobacterium smegmatis* arabinosyltransferase EmbC gene knockout strain). It exhibited some prominent cell wall defects, including tardiness of mycobacterial migration, loss of acid-fast staining, and increased cell wall permeability. Within alveolar epithelial cells (A549) infected by *M. sm*-ΔM_6387, the uptake rate was lower, phagosomes with bacterial degradation appeared, and microtubule-associated protein light chain 3 (LC3) recruitment was enhanced compared to wild type *Mycobacterium* *smegmatis* (*M. smegmatis*). We further confirmed that the variability in the removal capability of *M. sm*-ΔM_6387 resulted from host cell responses rather than the changes in the mycobacterial cell envelope. Moreover, we found that *M. sm*-ΔM_6387 or its glycolipid extracts significantly induced expression changes in some genes related to innate immune responses, including Toll-like receptor 2 (TLR2), class A scavenger receptor (SR-A), Rubicon, LC3, tumor necrosis factor alpha (TNF-α), Bcl-2, and Bax. Therefore, our studies suggest that nonpathogenic *M. smegmatis* can deposit LC3 on phagosomal membranes, and the decrease in the quantity of *Araf* residues for LAM molecules not only impacts mycobacterial cell wall integrity but also enhances host defense responses against the intracellular pathogens and decreases phagocytosis of host cells.

## 1. Introduction

Tuberculosis (TB) leads to millions of deaths each year [1,2]. Its prevalence largely depends on the ability of *Mycobacterium tuberculosis* (*M. tb*) to escape host immune surveillance [3]. *M. tb*, as an intracellular pathogen, interacts with phagocytes to trigger innate immune responses, whereas mycobacterial virulence factors can widely contribute to avoiding the protective immune responses [4,5,6,7].

Recently, many researchers have made efforts to develop host-directed therapy (HDT), which can better control TB by modulating host responses with or without additional antibodies [8,9,10]. We tried to explore a molecule that can evoke host defense responses against the pathogens, which are mainly determined by phagolysosomal pathways, including canonical autophagy and noncanonical autophagy [11,12]. The canonical autophagy pathway is characterized by autophagosome formation with a double-membrane, initiating microtubule-associated protein light chain 3 phospholipid conjugates (LC3-II) and phagophore formation in the vicinity of pathogens [13]. The noncanonical autophagy pathway includes LC3-associated phagocytosis (LAP), which is typically initiated by signaling through pathogen-recognition receptors (PPRs), such as Toll-like receptor 2 (TLR2), to promote LC3-II recruitment to single-membrane phagosomes [14,15,16]. LAP requires nicotinamide adenine dinucleotide phosphate (NADPH) oxidase and the phosphatidylinositol (PI)3-phosphate-kinase generated by the Rubicon–Beclin–Vps34 complex, which generates reactive oxygen species (ROS) and PI3-P to acquire LC3 on the phagosomes [17,18]. Rubicon (RUN domain protein as Beclin-interacting and cysteine-rich-containing), identified as a Beclin-1 binding partner, negatively regulates canonical autophagy and the endocytic pathway [19]. Importantly, it is required for LAP, located in the late phagosomes but not the autophagosomes [20,21]. *M. tb* can activate PPRs but does not robustly trigger LAP to eliminate mycobacteria within phagosomes [11,15]. Therefore, there is a close relationship between autophagy and phagocytosis. In contrast to autophagy, which contributes to the removal of intracellular pathogens, phagocytosis engulfs extracellular pathogens and plays a critical role in innate immune responses [22,23]. Recent studies revealed that phagocytosis was enhanced in autophagy-deficient macrophages, and autophagy might modulate phagocytosis [22,24,25].

The mycobacterial cell wall comprises peptidoglycan, arabinogalactan, and mycolic acids, along with lipopolysaccharides, polysaccharides, and proteins [26]. The lipopolysaccharides mainly consist of lipoarabinomannan (LAM), lipomannan (LM), and phosphatidylinositol mannosides (PIMs) [27]. LAM, located on the cell envelope, is a crucial immunomodulatory compound that participates in host–pathogen interactions [28,29]. There are three kinds of arabinosyltransferase in mycobacteria, namely, EmbA, EmbB, and EmbC, which catalyze the transfer of arabinofuranose (Ara*f*) residues to different substrate molecules. EmbC controls arabinan chain synthesis on an average of 55–70 *Araf* residues in LAM [30,31,32]. Importantly, the *Mycobacterium* genus represents a complex group of more than 100 species, some of which exhibit distinct LAM structures and elicit diverse host cell innate immune responses [28,33]. LAMs are mainly divided into two types—mannose-capped arabinomannan (manLAM) and PI-LAM. The manLAM from *M. tb* features mannanon at the terminus, and the PI-LAM without mannan at the terminus comes mainly from rapidly growing nonpathogenic strains such as *Mycobacterium smegmatis* (*M. smegmatis*) [33]. During host–pathogen interactions, LAMs exhibit a wide array of immunomodulatory activities, including cytokine effects, PPR interactions, and apoptosis induction [34]. The different terminal structures of the two LAM variants profoundly influence their biological activity [35]. ManLAM can block macrophage apoptosis, inhibit tumor necrosis factor alpha (TNF-α) and interleukin (IL)-12 production, induce IL-10 secretion, and limit TLR-mediated activation to weaken the cellular immune responses [36,37,38,39]. In contrast, PI-LAM may induce autophagy-related processes by TLR2 and activate the expression of pro-inflammatory cytokines [40,41]. Moreover, ManLAM suppresses the accumulation of LC3-II in phagosomes, whereas PI-LAM has no such effects [35]. Therefore, LAM diversity is responsible for innate immunity differences.

Some previous results suggest that EmbC gene knockout produces LAM-truncated variants with fewer *Araf* residues [32,42]. Reports indicated that LAM-truncated molecules with the shorter arabinan domains elicited stronger cytokine production responses, which are likely to be autophagy activators [38,43]. However, little is known about the effects of LAM-truncated variants on mycobacterial endocytosis, LC3 accumulation, phagocytosis, especially LAP. In this study, we first constructed a LAM-truncated mutant with fewer Ara*f* residues named *M. sm*-ΔM_6387, and wild type *M. smegmatis* as its parent strain; we further tested whether the structural variability in PI-LAM modulates autophagy-related processes, including phagocytosis and LAP.

## 2. Materials and Methods

### 2.1. Preparation of M. smegmatis EmbC Gene Knockout Strain

The *M. smegmatis* EmbC gene (MSMEG_6387), with its downstream sequence, was amplified using the forward primer (5′-GACTAGTCGGTCCGCATGCAGCGGGTGGCAGC-3′, *Spe*І) and the reverse primer (5′-TAGCGGCCGCTCAGCCGCTCAACTCAGCCGCAG-3′, *Not*І), using *M. smegmatis* mc^2^155 genomic DNA as a template. The target gene was amplified by PCR, and the product was cloned into the pMD18T vector to generate pMD18_M_6387. After DNA sequencing, pMD18_M_6387 was digested by *Nco*I to make blunt ends using Klenow DNA polymerase. Digested pUC4K by *Bam*HІ was used to obtain the kanamycin resistance cassette (Kan^R^, 1264 bp), and the Kan^R^ fragment was also filled in the blunt ends. The MSMEG_6387 gene was disrupted through the insertion of Kan^R^ to generate the pMD18_M_6387::Kan^R^ and pMD18_M_6387-kan^R^_i_ plasmids, and the pMD18_M_6387::Kan^R^ plasmid digested by *Spe*І and *Not*І was required to produce the M_6281::Kan^R^ fragment, and then the DNA fragment was cloned into pPR27-xylE to generate a conditional replication plasmid pPR27-M_6387::Kan^R^ (pJYІ) [44].

The pJYІ was electroporated into 100 μL *M. smegmatis*-competent cells under the condition of 2.5 Kv 1000 Ω with an electroporator 2510 (Eppendorf, Hamburg, Germany), and then the cells were grown in 2 mL Luria-Bertani (LB) medium with 0.05% Tween80 using 120 rpm shaking at 37 °C for 4 h. The cells were grown on LB agar plates containing Kan^R^ and Gen^R^ at 30 °C for approximately 4–5 d. Several clones were grown and picked from the LB agar plate containing Kan^R^ at 42 °C. When the first single-crossover event occurred, the mutants were stained yellow with 1% catechol. The positive clones were identified through PCR [45]. Transformants were grown on LB agar plates containing 25 μg/mL Kan and 10% sucrose at 37 °C to release the vector’s sequence by providing survival pressure. *M. sm*-ΔM_6387 produced with the second single-crossover event were then selected by PCR and verified by DNA sequencing. Furthermore, the expression levels of adjacent genes were identified through RT-PCR analysis, and sequences and conditions of the corresponding primers were showed in Table 1.

### 2.2. Extraction and Analysis of LAM/LM Mixture

Both wild type *M. smegmatis* and *M. sm*-ΔM_6387 were grown in 3 mL LB with 0.05% Tween80 (LBT) medium until the late logarithmic growth phase. Cells were harvested, washed with phosphate-buffered salt (PBS), and treated in 100 µL CHCL_3_:MeOH:H_2_O (20:20:3, *v*:*v*:*v*) at 55 °C for 30 min. The lysates were centrifuged at 14,000× *g* for 5 min, and the pellets were incubated with 200 µL Tris-saturated phenol: H_2_O (1:1, *v*:*v*) for 2 h at 80 °C. The samples were centrifuged at 14,000× *g* for 15 min after being mixed with 100 µL CHCL_3_, and the aqueous layer was transferred to a new Eppendorf (EP) tube to obtain the LAM/LM mixture [46]. The samples were analyzed on 15% SDS-PAGE through periodic acid-Schiff (PAS) staining. Western blotting was performed by transferring the antigens (LAM/LM) on SDS-PAGE gels to polyvinylidene difluoride (PVDF) membranes, and then the membrane was blocked and blotted with Concanavalin from *Canavalia ensiformis* peroxidase conjugate (Sigma-Aldrich, St. Louis, MO, USA), following visualization by Enhanced ECL (efficient chemiluminescence) kit (Wanleibio, Shenyang, China). The total glucose concentrations in the LAM/LM extraction were determined using the sulfate–phenol method.

### 2.3. Identification of Biological Characteristics

The mycobacterial growth curve was determined through the colony-forming units (CFUs). Briefly, *M. smegmatis* and *M. sm*-ΔM_6387 were collected at 6 h intervals and were diluted in PBS to generate 10-fold serial dilutions. Ten microliters of each diluted solution was spotted onto LB agar plates, repeated at least three times for all samples [46]. To observe the bacterial migration rates, *M. smegmatis* and *M. sm*-ΔM_6387 were (independently) spotted onto LB or LBT (LB with 0.05% tween 80) solid media and incubated at 37 °C until a bacterial ring was generated, and its diameter was measured [47]. To identify the integrity of the mycobacterial cell wall, *M. smegmatis* and *M. sm*-ΔM_6387 were cultured until reaching the logarithmic phase, and then acid-fast staining analysis was performed using the Ziehl–Neelsen method [46]. To identify cell wall permeability on chemical compounds, *M. smegmatis* and *M. sm*-ΔM_6387 at the logarithmic growth phase were serially diluted and treated with 0.5 μg/mL crystal violet and 0.005% SDS in triplicate, and then the CFU/mL values were determined [46]. Further electron microscopy analysis was also carried out according to the methods outlined previously [45].

### 2.4. Uptake and Survival of Intracellular Pathogens

A549 cells (Human type II alveolar epithelial cells) were grown in Dulbecco’s Modified Eagle Medium (DMEM) and supplemented with a 10% fetal bovine serum (FBS) and 1% penicillin–streptomycin mixture. A549 cells at a density of 8 × 10^3^ cells/mL were seeded in 96-well plates for 24 h. After being washed with PBS, the cells were infected by *M. smegmatis* or *M. sm*-ΔM_6387 strains, washed with PBST at a multiplicity of infection (MOI) value of 100, and cultured further using DMEM medium with 2% FBS at 37 °C for 6 h. Following incubation, cells were washed three times with PBS per well and treated by 100 μg/mL gentamicin for 1 h to remove extracellular bacteria [48]. Then, the cells were cultured and collected at specified time points (0, 6, 12, 24 h) to determine CFU aiming to measure the mycobacterial survival capability in A549 cells. A549 cells were also infected by *M. smegmatis*-FITC and *M. sm*-ΔM_6387-FITC at an MOI value of 100 for 6 h and removed extracellular bacteria according to the methods mentioned above; they were cultured further using DMEM medium with 10% FBS at 37 °C for 0, 6, and 24 h; and then washed and fixed with 4% paraformaldehyde for fluorescence microscopy. A549 cells were inoculated at a density of 1 × 10^5^ cells /well on 24-well plates and pre-treated with 100 μg/mL LAM/LM mixtures from *M. smegmatis* or *M. sm*-ΔM_6387 for 12 h, and then infected by *M. smegmatis* with a pSUM-kan-EGFP plasmid at an MOI of 100 for 6 h [49]. After removing extracellular bacteria and washing with PBST, the cells were incubated with rabbit polyclonal anti-TLR2 antibody (Abcam, Cambridge, UK) and a secondary antibody of Alexa Fluor 594-conjugated goat anti-rabbit IgG (Proteintech, Wuhan, China) and stained with DAPI for 5 min to observe the cells under a fluorescence microscope (SDPTOP ICX41, Ningbo, China).

### 2.5. Transmission Electron Microscopy (TEM)

All A549 cells treated or infected were centrifuged at 1200 rpm for 10 min, washed twice in PBS, and fixed by 2.5% glutaraldehyde overnight, followed by dehydration with gradient alcohol. Cells were embedded with epon and cut into ultrathin sections [45]. The sections were stained with 2% uranyl acetate for 30 min and lead citrate for 20 min, and the mitochondrial and phagosome structures within the A549 cells were then observed with a JEM-2000EX transmission electron microscope (JEOL, Tokyo, Japan). Finally, both mitochondria and phagosomes in the images were measured and counted using Image J (Version 2.6.1) software (NIH, Bethesda, MD, USA).

### 2.6. Detection of ROS Release in A549 Cells

To identify the release of ROS (reactive oxygen species) of A549 cells, the cells were treated by 100 μg/mL LAM/LM mixtures from *M. smegmatis* or *M. sm*-ΔM_6387 for 6, 12, or 24 h, and incubated with 20 μM DCFH-DA as a probe for 1 h for observation under a fluorescence microscope and measure relative fluorescence unit at 485/525 using ROS assay kit (Wanleibio, Shenyang, China).

### 2.7. Assessing LC3 Recruitments with a Confocal Fluorescence Microscope

To more directly measure LC-3 activation, we used a lentiviral vector with tandem-tagged RFP-GFP-LC3 (Genechem, Shanghai, China) to obtain stably transfected A549 cells (A549^+LC3^). Furthermore, A549^+LC3^ cells were infected by *M. smegmatis*, *M. sm*-ΔM_6387, BCG, or *M. smegmatis*::EGFP at an MOI of 100 for 6 h; cultured further using DMEM medium with 10% FBS at 37 °C for 1, 2, 3, or 48 h; and after washing, fixed with 4% paraformaldehyde for observation using a confocal fluorescence microscope(Leica TCS SP5ІІ, wetzlar, Germany). The images were processed and analyzed with Image J (version 2.6.1)software(NIH, Bethesda, MD, USA) [50].

### 2.8. The Expression Changes of Genes Related to Pathogen Recognition and Immune Responses

A549 cells were infected by mycobacteria or treated by LAM/LM mixtures and then collected at determined time point. Cell lysates were prepared using RIPA kit (WENLEI Biotech). Proteins were separated through 12% SDS-PAGE and transferred to PVDF membrane. The membrane was blocked with 5% skim milk powder and then incubated with a primary antibody including rabbit antibody against Beclin (Proteintech), Rubicon (Cell Signaling Tech., Danvers, IL, USA), LC3 (Proteintech, Wuhan, China), TLR2 (Abcam, Cambridge, UK), Bcl-2 (Proteintech, Wuhan, China), Bax (Proteintech, Wuhan, China), interferon gamma(IFN-γ) (Bioss, Beijing, China), and β-actin (Proteintech, Wuhan, China) at 4 °C overnight and a secondary antibody(anti-rabbit antibody, HRP conjugated, Proteintech) for 2 h. The bound probe was visualized by Enhanced ECL kit (WENLEI Biotech), and images were captured using a digital gel image processing system (Tanon 1600, Shanghai, China).

We picked up some genes related to pathogenic recognition and inflammatory responses and further detected their expression changes through qRT-PCR and RT-PCR. The corresponding primer sequences are listed in Table 2. Total RNA was extracted using Trizol (Sangon Tech, Shanghai, China.) A total of 1 μg RNA was first reversed into cDNA using RT kit (TaKaRa, Beijing, China), and then PCR was performed by corresponding cDNA as template; the ratios of the objected genes including TLR2, TLR4, Class A scavenger receptor (SR-A), Rubicon, IFN-γ, TNF-α, and IL-1β to internal control gene (β-actin) were used to assess the differences in gene expression levels.

### 2.9. Statistical Analysis

Data are presented as the mean ± standard error of the mean (SEM) of at least three independent experiments. All data were plotted and analyzed using GraphPad Prism version 8 (GraphPad software Inc., La Jolla, CA, USA) or Image J (version 2.6.1) software (NIH, USA). *p* < 0.05 was considered statistically significant (unpaired Student’s *t*-test). 

## 3. Results

### 3.1. Construction of an EmbC Gene Knockout Strain

**Figure 1:**
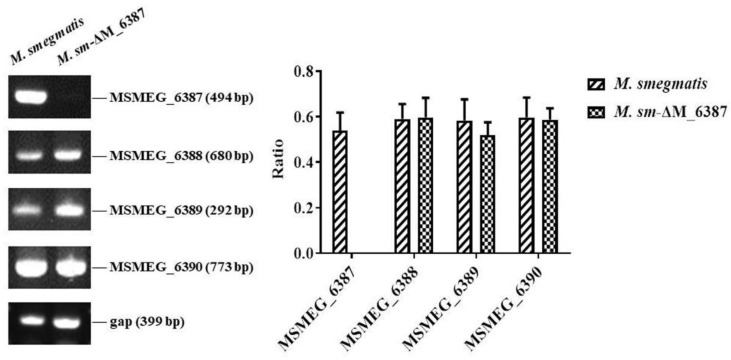
Identification of *M. smegmatis* EmbC gene knockout strain through RT-PCR.

EmbC gene knockout strains were constructed through a two-step homologous recombination technique similar to that outlined in our previous publication [45]. The identified mutant was further confirmed using RT-PCR analysis (Figure 1). Moreover, we confirmed a lack of MSMEG_6387 did not impact the expression levels of adjacent genes. Thus, the EmbC gene knockout mutant was successfully constructed and named *M. sm*-ΔM_6387.

### 3.2. EmbC Inactivation Induced the Change in LAM:LM in M. smegmatis 

**Figure 2:**
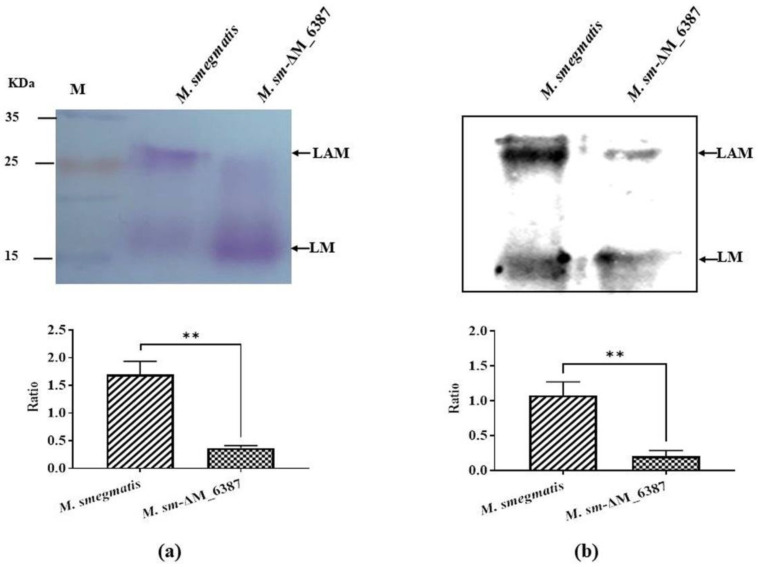
Separation and identification of lipopolysaccharides from the mycobacterial cell wall, including LAM and LM, and the double asterisks (**) represent *p* < 0.01. (**a**) PAS staining. (**b**) Western blotting analysis.

The ratio of Ara to Man in the cell wall provides useful information about its structural variability in relation to different biological functions [28,38]. Here, the cell wall lipopolysaccharides we extracted were a mixture, which included LAM and LM according to the molecular weight standard. We separated the LAM/LM mixture named PI-LAM/LM from wild type *M. smegmatis* and ΔLAM/LM from *M. sm*-ΔM_6387. The results showed that the LAM: LM value was 1.697 ± 0.139, and the ΔLAM:LAM value was 0.370 ± 0.025 using a PAS staining assessment (Figure 2a). The ratio of PI-LAM to LM was 1.077 ± 0.113, and the ratio of ΔLAM to LM was 0.207 ± 0.046 (Figure 2b). The results indicated that the LAM molecule of the mycobacterial cell wall was involved in structural variability due to the *M. smegmatis* EmbC gene knockout.

### 3.3. EmbC in M. smegmatis Is Critical for Mycobacterial Cell Wall Integrity

**Figure 3:**
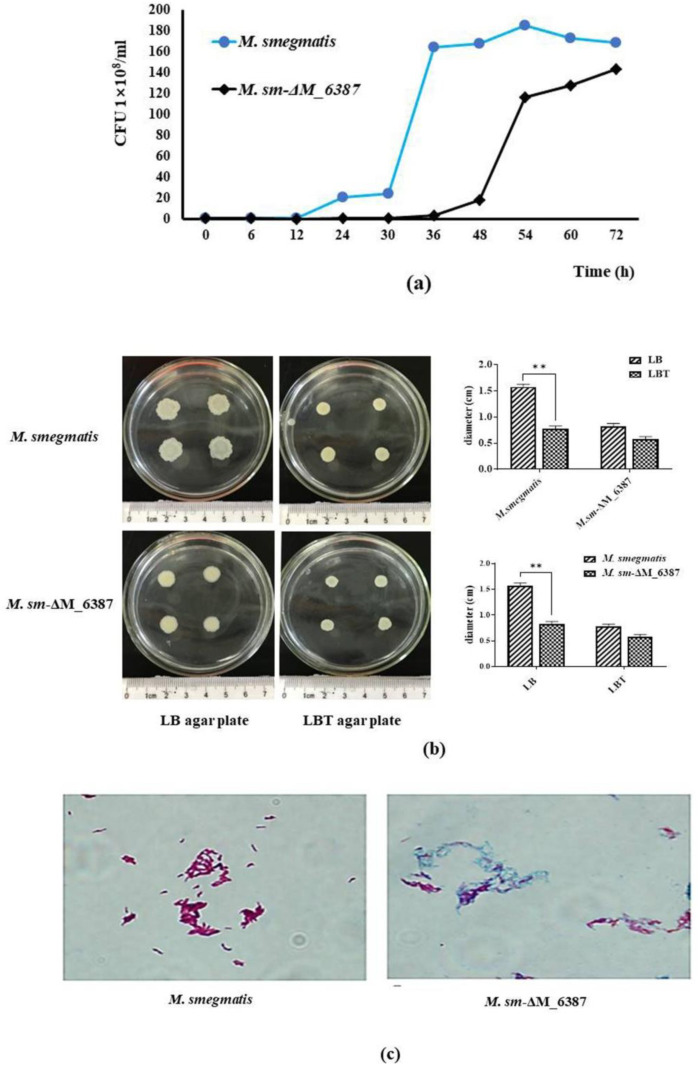

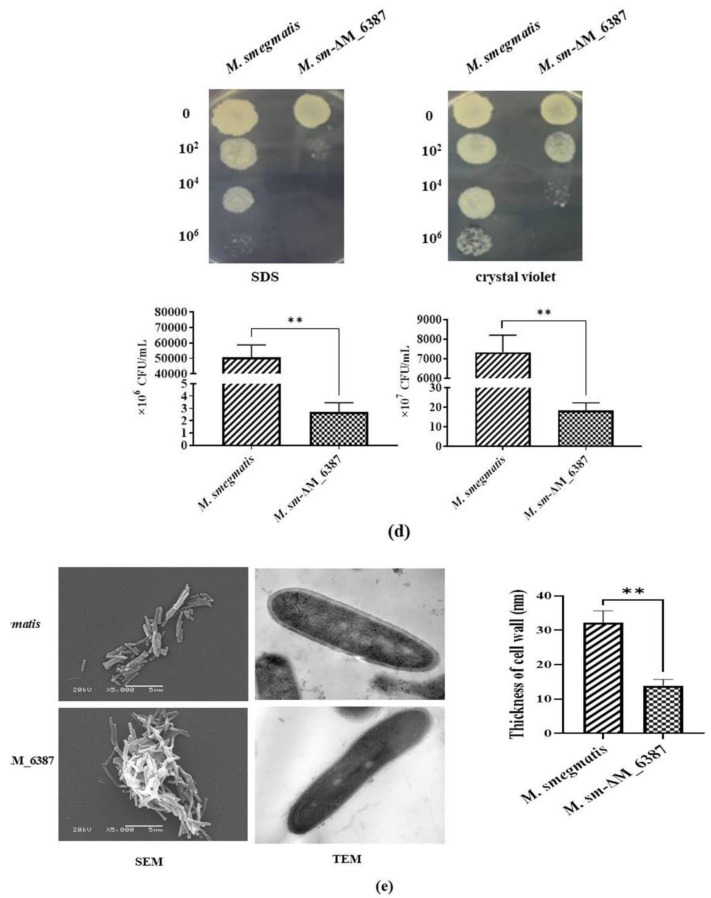
Assessment of the biological characteristics of *M. smegmatis* and *M. sm*-ΔM_6387. (**a**) Growth curves of *M. smegmatis* and *M. sm*-ΔM_6387. (**b**) Measurement of the migration rate through colony diameter on different LB agar plates. (**c**) Acid-fast staining analysis using the Ziehl–Neelsen method. (**d**) Detection of cell wall permeability through SDS and crystal violet. (**e**) Morphology and cell wall structures of *M. smegmatis* and *M. sm*-ΔM_6387 were observed by SEM (5000×) and TEM (8000×), respectively. The double asterisks (**) represent *p* < 0.01.

The CFU results indicated that the growth of *M. sm-ΔM_6387* was tardy compared to *M. smegmatis*, particularly at the early logarithm stage, but the growth of *M. sm*-ΔM_6387 was not completely inhibited by EmbC inactivation (Figure 3a). Therefore, *M. smegmatis* EmbC is a non-essential gene for growth. We also found that *M. sm*-ΔM_6387 strains had a smoother cell envelope and lower bacterial migration rate (Figure 3b). Acid fastness is a characteristic used to detect mycobacterial cell wall integrity, and cell walls lacking integrity cannot be stained. We found that *M. smegmatis* was a typical red following carbol-fuchsin staining, whereas most of the *M. sm*-ΔM_6387 were stained blue (Figure 3c). We also measured the sensitivity of the cell wall to chemical compounds using crystal violet and SDS. These hydrophilic compounds are used to test plasma membrane permeability. We found that *M. sm*-ΔM_6387 was sensitive under the selective pressure of crystal violet and SDS compared with *M. smegmatis*, and the mutant strain had higher permeability to the hydrophilic compounds (Figure 3d). These results suggest that *M. smegmatis* EmbC inactivation inhibits mycobacterial migration while increasing cell wall permeability, most likely due to a more hydrophilic cell envelope.

Another observation was found that there was a bulge in the middle and at the ends of *M. sm*-ΔM_6387 through SEM. Furthermore, TEM results indicated that *M. sm*-ΔM_6387 was thinner (13.91 ± 1.85 nm) in cell wall width compared to *M. smegmatis* (32.14 ± 3.52 nm; Figure 3e). These results further demonstrate that the roles of EmbC are related to mycobacterial cell wall integrity.

### 3.4. Intracellular Uptake of M. smegmatis and M. sm-ΔM_6387 

**Figure 4:**
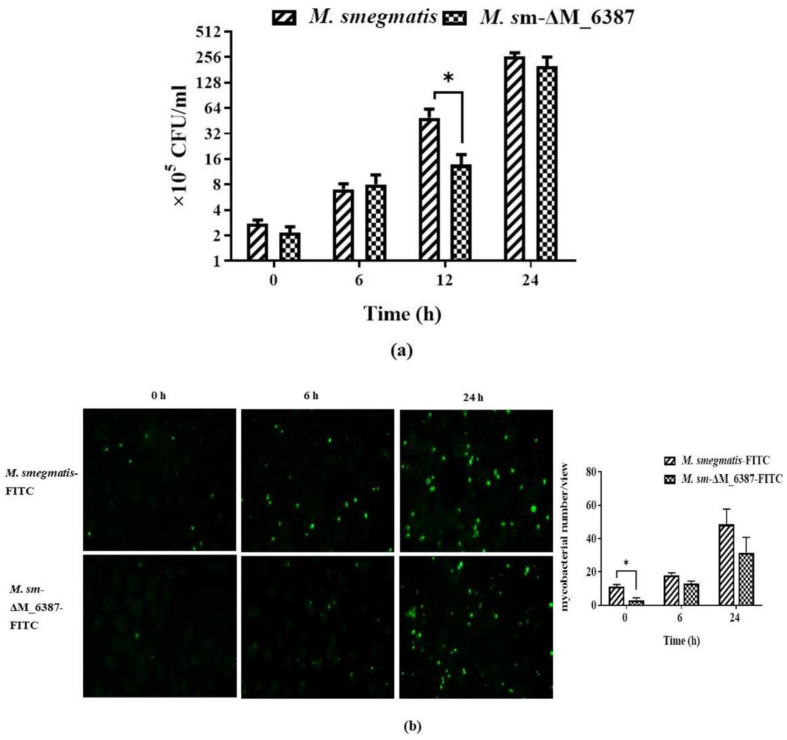

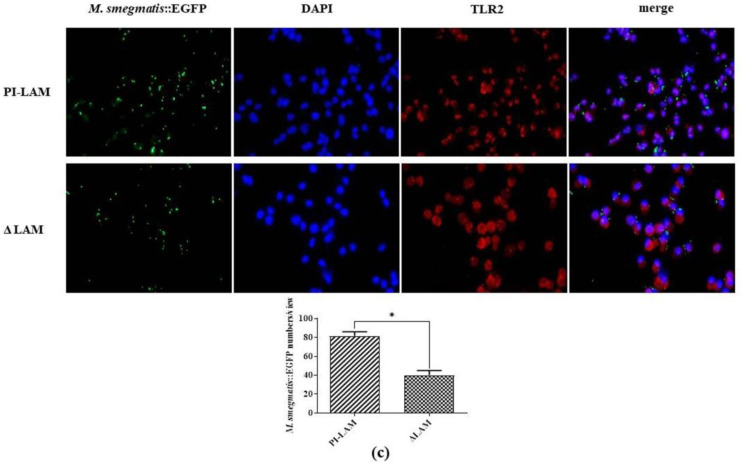
Survival and persistence of *M. smegmatis* and *M. sm*-ΔM_6387 in A549 cells. (**a**) CFU over time post-infection. (**b**) Observations of internalized *M. smegmatis*-FITC and *M. sm*-ΔM_6387-FITC in A549 cells under a fluorescence microscope (400×). (**c**) Reproduction of *M. smegmatis*::EGFP internalized in A549 cells after PI-LAM/LM and ΔLAM/LM treatment (400×), and the single asterisk (*) represents *p* < 0.05.

The mycobacteria internalized in A549 cells were assessed by CFU curves. *M. sm*-ΔM_6387 was found to have a significantly lower reproductive rate compared to *M. smegmatis* at 12 h post-infection (Figure 4a). We also observed internalized *M. smegmatis*-FITC and *M. sm*-ΔM_6387-FITC in A549 cells, and the uptake of *M. sm*-ΔM_6387-FITC was decreased after infection (Figure 4b). We wanted to understand whether the differences in phagocytosis between *M. smegmatis* and *M. sm-ΔM_6387* result from the structural differences in the mycobacterial cell wall or from the responses of host innate immunity. Hence, A549 cells were pre-treated with PI-LAM/LM or ΔLAM/LM for 12 h and then infected by the same *M. smegmatis*::EGFP strain to detect mycobacterial uptake rate. The findings indicate that ΔLAM/LM resulted in a lower mycobacterial uptake rate than PI-LAM/LM (Figure 4c). Hence, ΔLAM/LM from *M. sm*-ΔM_6387 hampered mycobacterial uptake and appeared to induce more defense responses against pathogens, potentially contributing to the removal of the intracellular pathogen.

### 3.5. Changes in the Phagosome and Mitochondrial Size within Infected Alveolar Epithelial Cells

**Figure 5:**
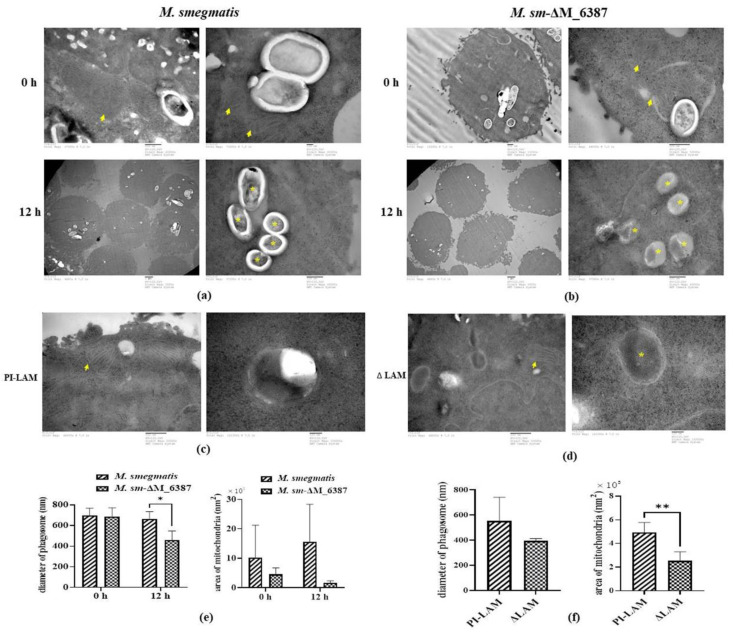
TEM analysis of A549 cells. (**a**) A549 cells infected by *M. smegmatis* at 0 or 12 h post-infection; (**b**) A549 cells infected by *M. sm*-ΔM_6387 at 0 or 12 h; (**c**) A549 cells treated by PI-LAM from *M. smegmatis*; (**d**) A549 cells treated by ΔLAM from *M. sm*-ΔM_6387; (**e**) The mean sizes of phagosomes and mitochondria in infected A549 were respectively analyzed, and the asterisks (*) represents *p* < 0.05; (**f**) The mean sizes of phagosomes and mitochondria in A549 cells pre-treated by LAM mixture were showed, and the double asterisks (**) represent *p* < 0.01. Red arrows point to all kinds of mitochondria, and the yellow asterisks are exhibited within phagosomes untaked bacteria.

To investigate the defense responses in infected A549 cells, the ultrastructure of host cells engulfing pathogens was observed through TEM. We found that individual A549 cells contained different numbers of phagosome-engulfed bacteria at 0 and 12 h post-infection. The mean diameter of phagosome-engulfed *M. smegmatis* at the primary stage of infection was 697 ± 70 nm, and that of *M. sm*-ΔM_6387 was 688 ± 83 nm, the difference between which is not significant. At 12 h post-infection, the mean diameter of phagosomes within A549 cells infected by *M. smegmatis* was 664 ± 60 nm, whereas the cells infected by *M. sm*-ΔM_6387 contained many compartments exhibiting bacterial degradation that were 458 ± 24 nm in diameter, which were likely to be LAPosomes. The LAPosomes of the latter were significantly smaller in size than the phagosomes of the former (Figure 5a,b). We also found that several mitochondria in A549 cells infected by *M. smegmatis* were enlarged over five times in size compared to ones infected by *M. sm*-ΔM_6387 at 12 h post-infection (Figure 5a,b).

To further illustrate whether LAM molecules directly affect the role of mitochondria, LAM extraction was used to treat A549 cells. Our results indicate that PI-LAM from *M. smegmatis* also elicited larger-sized mitochondria than ΔLAM/LM extraction from *M. sm*-ΔM_6387 (Figure 5c,d). These results suggest that both *M. smegmatis* and *M. sm*-ΔM_6387 can trigger the maturation of phagosomes, but *M. sm*-ΔM_6387 appeared to have poorer survival due to degradation in the observed cells. In addition, PI-LAM from nonpathogenic *M. smegmatis* might induce stronger reactions in mitochondria compared to ΔLAM from *M. sm*-ΔM_6387. 

### 3.6. LAM/LM Molecules Affected the Release of ROS

**Figure 6:**
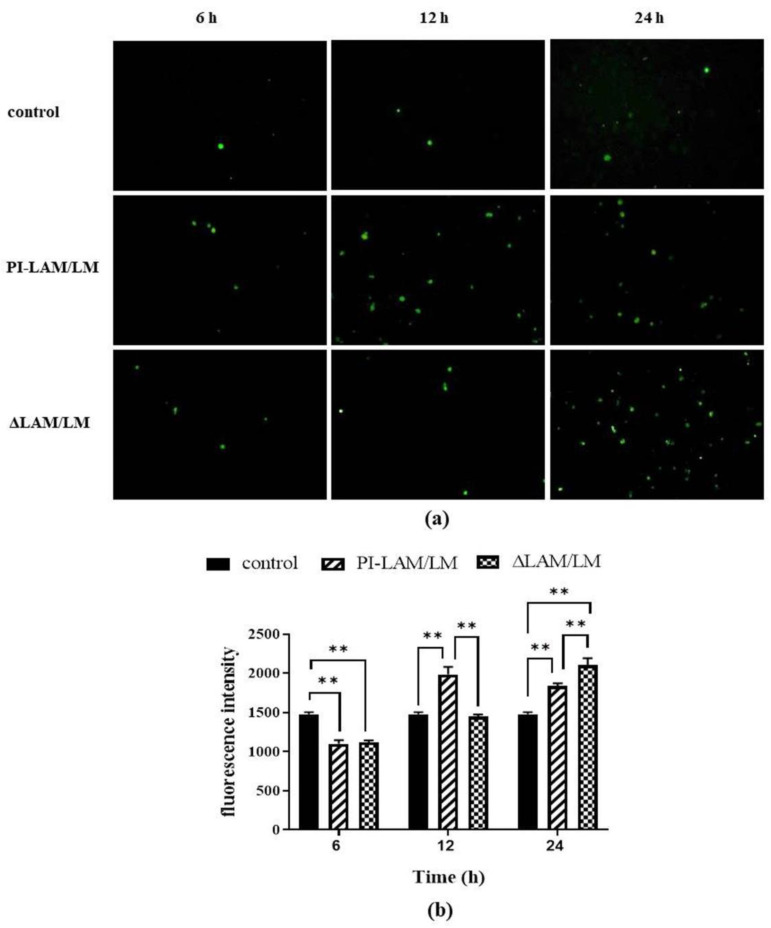
A549 cells were treated by PI-LAM/LM and ΔLAM/LM at 37 °C for 6, 12, or 24 h and loaded with DCFH-DA probes in order to detect the release of ROS by fluorescence microscopy (**a**) and quantitative detection with a multifunctional microplate reader, and the double asterisks (**) represent *p* < 0.01 (**b**).

ROS can promote the permeability of the mitochondrial membrane and increase the release of Ca^2+^, and so they play a critical role in the induction of apoptosis mediated by the mitochondrial pathway [51]. In addition, LAP depends on ROS generated by NADPH oxidase, which can directly kill bacteria [18]. Our results indicated that ROS released from the two LAM/LM treatment groups gradually increased with the duration of LAM/LM treatment. PI-LAM/LM treatment induced ROS release at 12 h, whereas ΔLAM/LM treatment activated ROS release at 24 h (Figure 6). Thus, LAM from *M. smegmatis* can enhance ROS release, which is probably a critical factor to induce intracellular pathogen clearance, and a decrease in Ara*f* residues in LAM molecules may modulate ROS release.

### 3.7. Recruitment and Localization of LC3 Molecules within Infected Alveolar Epithelial Cells

**Figure 7:**
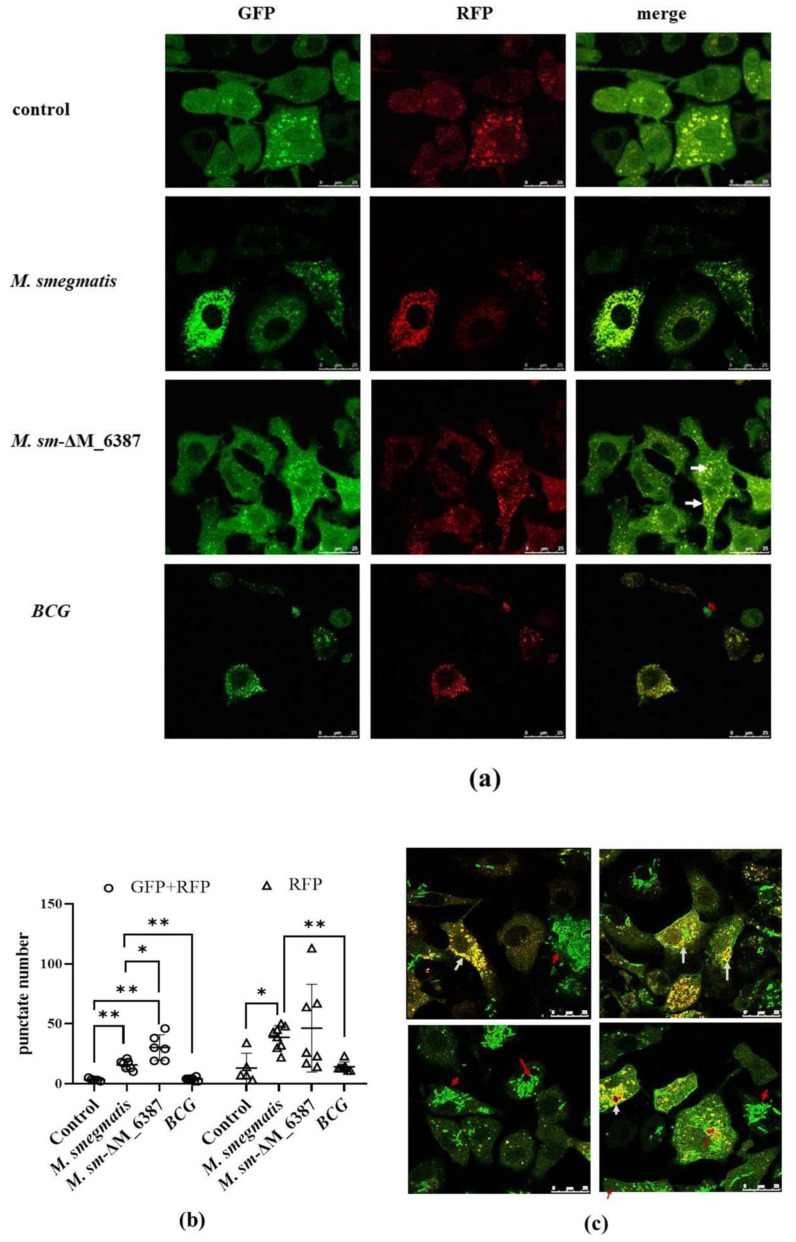
Confocal images of A549 cells transient transfected by a lentiviral vector with tandem-tagged RFP-GFP-LC3 (A549^+LC3^) post-infection. (**a**) A549^+LC3^, respectively, infected by *M. smegmatis*, *M. sm*-ΔM_6387, and BCG for 48 h, and control group represent untreated A549 ^+ LC3^ cells (1260×); and white arrows point to LAPsome structures. (**b**) Dot plots showing yellow and red spot numbers within A549^+LC3^ cells treated by different mycobacteria; circles indicate the number of yellow punctates which mean LC3 recruitments, and triangles represent the number of red punctates. (**c**) A549^+LC3^ infected by *M. smegmatis* or *M. smegmatis*-EGFP; white arrows indicate LC3-positive recruitment, and red arrows point to intracellular *M. smegmatis*-EGFP (1260×). The single asterisk (*) represents *p* < 0.05, and the double asterisks (**) represent *p* < 0.01.

To further evaluate LC3-mediated phagocytosis compartments, we used a tandem-tagged RFP-GFP-LC3 vector to construct A549^+LC3^ cells. Due to the differential pH sensitivity of GFP and RFP, non-acidic phagocytosis compartments are labeled with both GFP and RFP; however, the phagolysosome, after fusing with a lysosome, becomes acidic, and GFP fluorescence is quenched, and only RFP remains [41]. Here, we found that both *M. smegmatis* and *M. sm*-ΔM_6387 caused a significant increase in non-acidic yellow spots (RFP + GFP) 2 h post-infection. *M sm*-ΔM_6387 had a particularly large number of spots; however, BCG with man-LAM did not cause a specific change in yellow spot number (Figure 7a,b and Figure S1). Moreover, EGFP molecules seemed to be embraced on the cell membrane, while LC3 recruitment was obvious, which might have resulted from the leakage of the *M. smegmatis*::EGFP strains being killed (Figure 7c). The data indicate that both *M. smegmatis* and *M sm*-ΔM_6387 are able to induce LC3 recruitment, but BCG strains do not. 

### 3.8. LAM/LM Molecules Regulated Gene Expression Related to Pathogenic Recognition and Immune Responses

**Figure 8:**
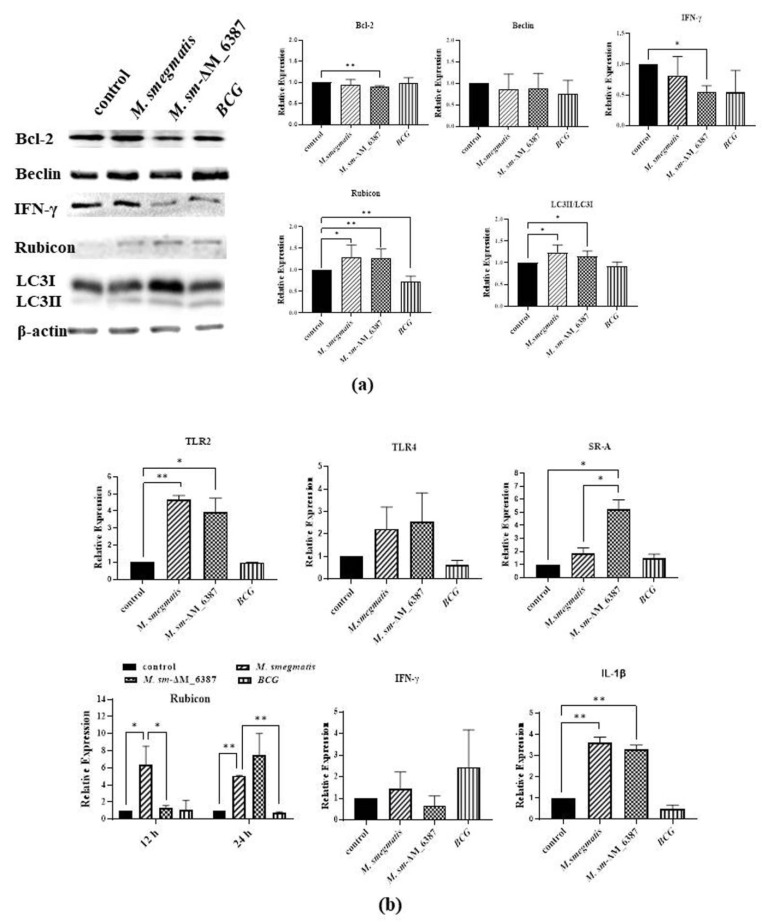

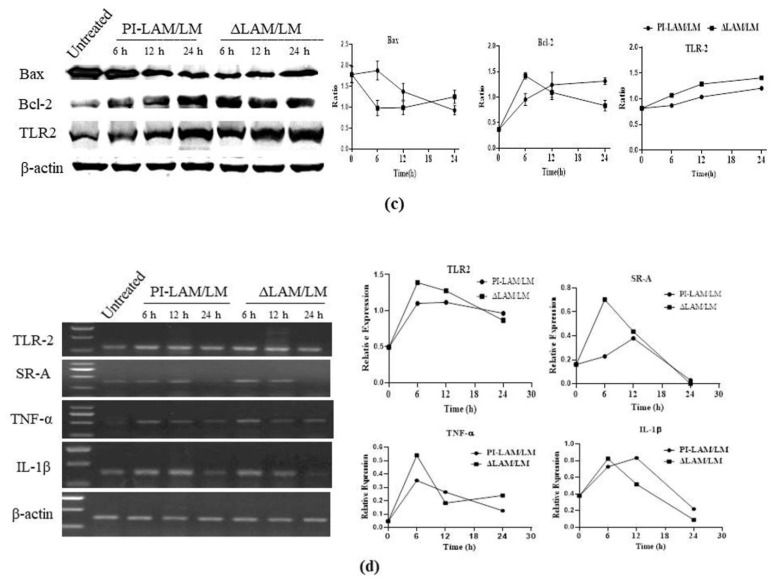
The identification of the expression levels of proteins or genes related to LAP and inflammatory responses through Western blotting and RT-PCR. (**a**) A549 cells were infected by *M. smegmatis*, *M. sm*-ΔM_6387, or BCG for 6 h, and then were collected 12 h post-infection for the identification of protein expression changes through Western blotting. The proteins analyzed included Bcl-2, Beclin, Rubicon, IFN-γ, and LC3, and the internal reference, β-actin. The significant differences in expression are marked. (**b**) qRT-PCR was used to detect mRNA expression, and the genes analyzed included TLR2, TLR4, SR-A, Rubicon, IFN-γ, and IL-1β. (**c**) A549 cells were treated PI-LAM/LM or ΔLAM/LM and were incubated at 37 °C for 6, 12, or 24 h. The expression changes of three genes, Bax, Bcl-2, and TLR2, were identified through Western blotting. (**d**) The expression changes of four genes, TLR2, SR-A, TNF-α, and IL-1β, were identified through RT-PCR analysis. The single asterisk (*) represents *p* value < 0.05, and statistical difference, and the double asterisks (**) represent *p* value < 0.01, and significance difference.

A549 cells were collected at 12 h post-infection to detect the expression changes of genes related to LAP, including Bcl-2, Beclin, Rubicon, IFN-γ, and LC3, through Western blotting. The results indicated that Rubicon was upregulated in both *M sm*-ΔM_6387 and *M. smegmatis* compared with the control group; the lipidated LC3 II was also upregulated in the two strains. In addition, we also found that *M sm*-ΔM_6387 significantly downregulated the expression of IFN-γ compared with the control group (Figure 8a). The decrease in IFN-γ expression is likely to relate to a lesser intercellular mycobacterial load. The QRT-PCR results exhibited that *M sm*-ΔM_6387 upregulated the expression of SR-A, Rubicon, and IL-1β. In particular, Rubicon expression was obviously increased at 24 h post-infection (Figure 8b). It is necessary to clarify the mechanisms of the expression changes of IFN-γ and SR-A in the future.

Further, we treated A549 cells using LAM/LM extraction and detected the expression changes of genes related to innate immunity. The results indicated that Bax was gradually downregulated and Bcl-2 was gradually upregulated over the course of PI-LAM/LM treatment, whereas the opposite tendencies were seen in the ΔLAM/LM treatment group. Additionally, the expression of TLR2 was significantly enhanced after ΔLAM/LM treatment (Figure 8c).

RT-PCR results exhibited that SR-A and TLR2 were significantly upregulated, along with inflammatory cytokines (TNF-α and IL-1β) at 6 h after ΔLAM/LM treatment (Figure 8d).

## 4. Discussion

After *M. tb* infection, LAM can modulate the innate immunity of phagocytes, such as alveolar epithelial cells, macrophages, and dendritic cells [52,53]. The innate immune responses against mycobacteria are mainly involved in inflammatory factors secretion, phagocytosis, autophagy, and bactericidal activity [20,54]. LAM is recognized through mannose receptor (MR), dendritic cell-specific intercellular adhesion molecule-3-grabbing non-integrin (DC-SIGN), and TLRs [55]. SR-A has been demonstrated to modulate TLRs signaling [56]. LAM possesses diversity receptor affinity and heterogeneous immunogenicity due to the differences in LAM structures [57].

This study first exhibited that *M. sm*-ΔM_6387 with LAM-truncated molecules had some prominent cell wall defects and migration inhibition. The changes suggest that the cell envelope of *M. sm*-ΔM_6387 tends to have a higher permeability compared with wild type *M. smegmatis*. When carrying out the studies on the phagocytosis of alveolar epithelial cells (A549), we found that *M. sm*-ΔM_6387 had lower intracellular uptake and retarded growth compared to *M. smegmatis*. We wondered whether the consequences were determined by mycobacterial biological characteristics such as the cell envelope permeability and mycobacterial migration or different immunity of alveolar epithelial cells. Here, we confirmed that the effects did not result from mycobacteria but the divergent reactions of host cells through the studies on the same mycobacteria to challenge A549 cells pre-treated by LAM.

Further studies found that both *M. smegmatis* and its variant *M. sm*-ΔM_6387 deposited LC3 on phagosomal membranes. Moreover, the levels of their LC3 recruitment were significantly different. We observed that *M. sm*-ΔM_6387 increased mycobacterial degradation in phagosomes in TEM images. The results suggest that *M. sm*-ΔM_6387 induces a stronger removal ability of intracellular bacteria, which is likely due to the regulation of LAP.

Rubicon is an essential and positive regulator of the NADPH oxidase complex, which activates LAP [18,19,22]. Our results indicated that both *M. smegmatis* and *M. sm*-ΔM_6387 upregulated Rubicon expression; moreover, *M. sm*-ΔM_6387 induced stronger Rubicon mRNA expression at 24 h post-infection. In addition, the two strains also enhanced, transiently, and mRNA expression of genes related to protective immunity, including TLR2, SR-A, and IL-1β. Interestingly, we also found that ΔLAM/LM from *M. sm*-ΔM_6387 can strongly upregulate mRNA expression of TLR2, SR-A, TNF-α at 6h, and enhance ROS release at 24 h. Therefore, the findings suggest that the LAM–truncated molecules differently influence innate immune responses against intracellular bacteria, which is likely to be related to LAP.

The studies indicated that the clearance ability of host cells for a pathogen was prominently determined by autophagy [16,58]. Previous studies also found that nonpathogenic *M. smegmatis* within alveolar epithelial cells may be eliminated or replicated, mainly depending on the intracellular mycobacterial load and the effectiveness of the immune responses [11,59,60,61]. On the basis of TLR2 activation, Rubicon interacts with one subunit of the NADPH oxidase complex, enhancing phagosomal trafficking to induce ROS release and the expression of inflammatory cytokines to defend against invaders [18,19]. Our results demonstrated that nonpathogenic *M. smegmatis* could activate the expression of genes related to the LAP pathway, and the arabinose domain of PI-LAM modulates the activation.

In addition, we found that ΔLAM/LM from *M. sm*-ΔM_6387 decreased mycobacterial uptake in our experimental conditions. Another interesting observation is that individual phagocyte has distinct uptake ability in TEM and confocal images, being likely to elicit divergent phagocytosis. Moreover, mycobacterial uptake ability is contrary to LC3 activation. The findings led us to hypothesize that LAP inhibits mycobacterial phagocytosis and intracellular survival. SR-A, with a wide range of ligand targets, is expressed on phagocytes. In the lungs, SR-A mediates uptake and pathogenic clearance and has critical roles in the capture and degradation of pathogens [25,62]. These results demonstrate that the LAM can better activate SR-A mRNA expression. Furthermore, the time-dependent expression changes of Bcl-2 caused by ΔLAM/LM and PI-LAM/LM were opposite. As we know, Beclin-1 is essential for LAP, and the Beclin-1/VPS34 complex is repressed by Bcl-2 [20]. It is an interesting question whether the expression changes of Bcl-2 affect the activation of Beclin-1 during LAM treatment. However, we did not check the statistical difference of Beclin-1 expression in this study.

Taken together, nonpathogenic *M. smegmatis* can deposit LC3 on phagosomal membranes, and the decrease in the quantity of *Araf* residues of LAM molecules not only impacts mycobacterial cell wall integrity but also enhances host defense responses against devastating pathogens. Given LAM-truncated molecules could be used as a tool for the development of HDTs, it is important to resolve how circulating LAM in serum enters the host cell cytoplasm [43,63]. Our results indicate that both PI-LAM and the variant ΔLAM are of interest to the modulation of innate immune responses. These results provide a clue to uncovering the relationship between the structure of LAM and innate immune responses. On the basis of these findings, it is necessary to further explore the molecular mechanisms in detail.

## Figures and Tables

**Table 1:** Sequences and conditions of the mentioned primers related to *M. smegmatis*.

Gene	Primer Sequences (5′–3′)	Annealing Temperature	Product Length (bp)
MSMEG_6387	F: CCGACCCTGCTGAAACTGCT	58	494
	R: AGCCAGAACGCCAGGAACAG		
MSMEG_6388	F: GTCGGTGCGCATCAAGTACG	58	680
	R: CCTTGACCATCGAGCCGAGT		
MSMEG_6389	F: CGATCGTGTCGACCGTCATC	58	292
	R: ATGAGCGCCAGCACGTTGTA		
MSMEG_6390	F: CTGGCTGGTGCTCGAACTCA	58	773
	R: CCAGCACACATCCGTTGAGG		
gap	F: GGAAAGCTGTGGCGTGATGG	54	399
	R: GTAGGCCATGAGGTCCACCA		

**Table 2:** Sequences and conditions of the mentioned primers related to A549 cells.

Gene	Primer Sequences (5′–3′)	Product Length (bp)
TLR-2	F: GGAATCGGTGAGGTCCTGTCCTG	296
	R: GGCGTCACATGCAGAAAGCCC	
TLR-4	F: AAGTGTCTGAACTCCCTCCAGG	278
	R: ATGGTCTTATTCATCTGACAGGTGATA	
SR-A	F: ATGTCCGTTCAGCGTCTT	380
	R: TAGGTCCTGATGCTTCTTTA	
Rubicon	R: AGTGGGTTACTTGGGAGTG	310
	F: CTTTGGCTAATAGTTCTGC	
IFN-γ	F: TCGGTAACTGACTTGAATGTCCA	93
	R: TCGCTTCCCTGTTTTAGCTGC	
IL-1β	F: GGACAAGCTGAGGAAGATGC	360
	R: TCTTTCAACACGCAGGACAG	
TNF-α	F: TGCTTGTTCCTCAGCCTCTT	514
	R: GGAAGACCCCTCCCAGATAG	
β-actin	F: CATGGATGATGATATCGCCGCG	371
	R: ACATGATCTGGGTCATCTTCTCG	

## Data Availability

The A549 cell line was provided by national collection of authenticated cell cultures. The plasmids relating to the construction of *M. smefmatis* geneknock out strain were obtained from Prof. Yufang Ma, and the pSUM-kan-EGFP reporter plasmid was obtained from Dr. Nicolai S.C. van Oers.

## Supplementary Materials

The following supporting information can be downloaded at: https://www.mdpi.com/article/10.3390/cells11030361/s1. Figure S1: Confocal image of A549 cells transiently transfected by a lentiviral vector with tandem-tagged RFP-GFP-LC3 (A549^+LC3^) post-infection. (a) A549^+LC3^ infected by *M. smegmatis*, *M. sm*-ΔM_6387, or BCG for 1, 2, and 3 h, respectively (630×); white arrows point to LAPsome structures. (b–d) Dot plots showing yellow and red spot numbers within A549^+LC3^ cells treated by different mycobacteria; circles indicate the number of yellow punctates, and triangles point to red spots; the panels of b, c and d showed respectively 1, 2, and 3 h post-infection; the asterisks including *, **, *** and **** represents *p* < 0.05 or lower.
