# Supplementary material for: Structural Variability of Lipoarabinomannan Modulates Innate Immune Responses within Infected Alveolar Epithelial Cells
Source: Cells. 2022 Jan 21;11(3):361. doi: 10.3390/cells11030361 (PMC8834380; doi:10.3390/cells11030361)
Supplement: Supplementary file 1 [file cells-11-00361-s001.zip › cells-1528074-supplementary.pdf]

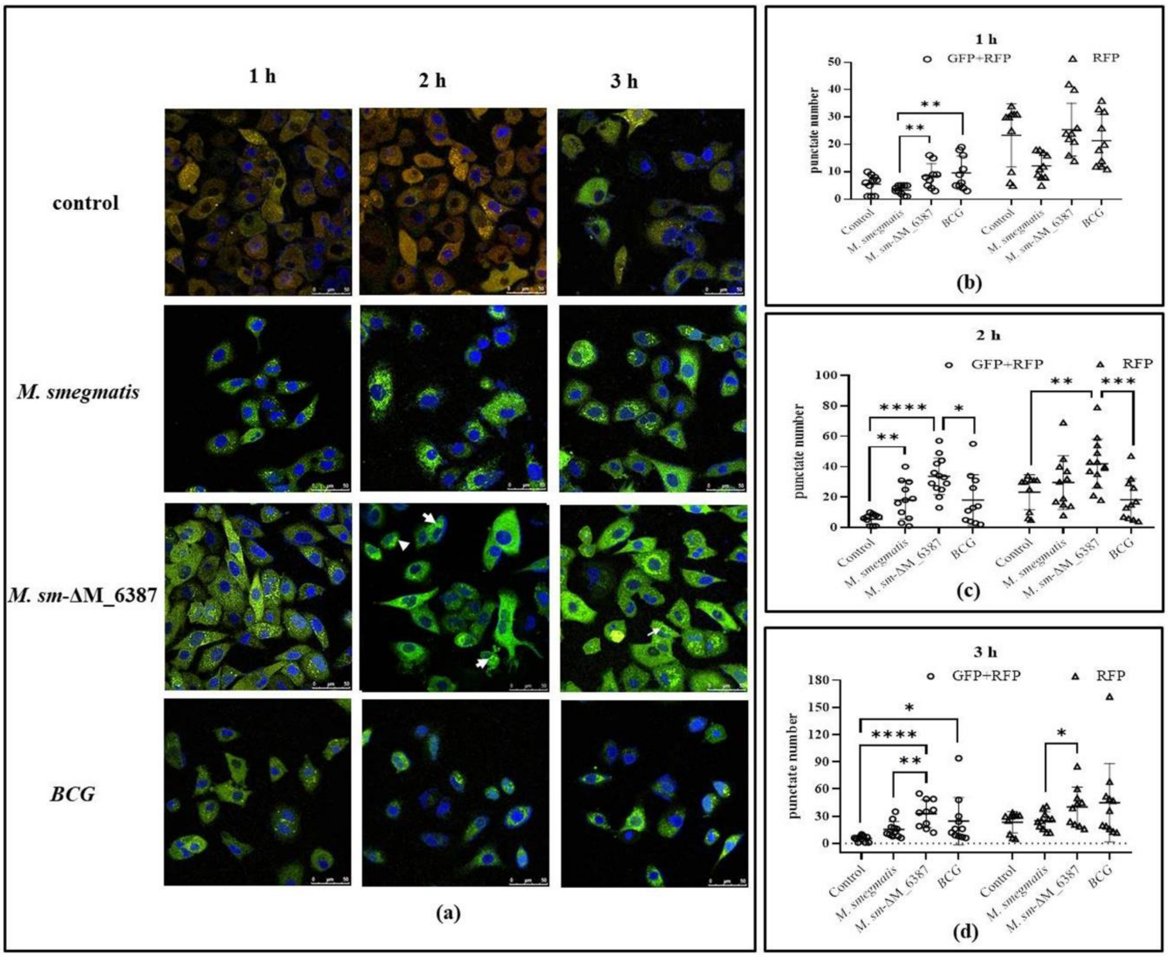

Figure S1: Confocal image of A549 cells transiently transfected by a lentiviral vector with tandem-tagged RFP-GFP-LC3 (A549^{+LC3}) post-infection. (**a**) A549^{+LC3} infected by *M. smegmatis*, *M. sm-ΔM_6387*, or BCG for 1, 2, and 3 h, respectively(630×); white arrows point to LAPsome structures. (**b**-**d**) Dot plots showing yellow and red spot numbers within A549^{+LC3} cells treated by different mycobacteria; circles indicate the number of yellow punctates, and triangles point to red spots; the panels of b, c and d showed respectively 1, 2, and 3 h post-infection; the asterisks including *,**, *** and ***** represents $p < 0.05$ or lower.
